# Automated muscle histopathology analysis using CellProfiler

**DOI:** 10.1186/s13395-018-0178-6

**Published:** 2018-10-18

**Authors:** Yeh Siang Lau, Li Xu, Yandi Gao, Renzhi Han

**Affiliations:** Department of Surgery, Davis Heart and Lung Research Institute, Biomedical Sciences Graduate Program, Biophysics Graduate Program, The Ohio State University Wexner Medical Center, Columbus, OH 43210 USA

**Keywords:** Histology, Muscle, Image segmentation, Quantitative analysis, *mdx* mouse

## Abstract

**Background:**

Histological assessment of skeletal muscle sections is important for the research of muscle physiology and diseases. Quantifiable measures of skeletal muscle often include mean fiber diameter, fiber size distribution, and centrally nucleated muscle fibers. These parameters offer insights into the dynamic adaptation of skeletal muscle cells during repeated cycles of degeneration and regeneration associated with many muscle diseases and injuries. Computational programs designed to obtain these parameters would greatly facilitate such efforts and offer significant advantage over manual image analysis, which is very labor-intensive and often subjective. Here, we describe a customized pipeline termed MuscleAnalyzer for muscle histology analysis based upon CellProfiler, a free, open-source software for measuring and analyzing cell images.

**Results:**

The MuscleAnalyzer pipeline consists of loading, adjusting, and running a series of image-processing modules provided by CellProfiler. This pipeline was evaluated using wild-type and *mdx* muscle sections co-stained with laminin (to demarcate the muscle fiber boundaries) and 4′,6-diamidino-2-phenylindole (DAPI, to label the nuclei). The immunofluorescence images analyzed using the MuscleAnalyzer pipeline or manually yielded similar results in the number of muscle fibers per image (*p* = 0.42) and central nucleated fiber (CNF) percentage (*p* = 0.29) in *mdx* mice. However, for a total of 67 images, CellProfiler completed the analysis in ~ 10 min on a regular PC while it took an investigator ~ 3 h using the manual approach in order to quantify the number of muscle fibers and CNF. Moreover, the MuscleAnalyzer pipeline also provided the measurement of the cross-sectional area (CSA) and minimal Feret’s diameter (MFD) of muscle fibers, and thus fiber size distribution can be plotted.

**Conclusions:**

Our data indicate that the MuscleAnalyzer pipeline can efficiently and accurately analyze laminin and DAPI co-stained muscle images in a batch format and provide quantitative measurements for muscle histological properties such as muscle fiber diameters, fiber size distribution, and CNF percentage.

**Electronic supplementary material:**

The online version of this article (10.1186/s13395-018-0178-6) contains supplementary material, which is available to authorized users.

## Background

Skeletal muscle is an exceptionally adaptive tissue. During endurance exercise, skeletal muscle undergoes extensive adaptation by changing their fiber type composition and fiber size [1–3]. Upon injuries, satellite cells associated with skeletal muscle are activated to proliferate, fuse to form myotubes, and eventually regenerate new muscle fibers [4–7]. In genetic myopathies such as Duchenne muscular dystrophy (DMD), a fatal X-linked recessive muscle disease caused by genetic mutations leading to the loss of dystrophin [8], repeated cycles of muscle injury, and repair result in increased variation of fiber size and muscle fibers with central nuclei [9, 10]. Examination of muscle cross-sections is therefore often carried out to assess such changes in the fields of myopathy and rehabilitation science. However, the methods to quantify these changes remain challenging among investigators and often require painstaking manual procedures [11, 12]. Traditionally, visually identifying muscle nuclei and manually measuring the muscle fiber size manual tracing of individual fibers are relatively subjective and time consuming. These tasks are highly susceptible to both inter-individual and inter-laboratory variability, often resulting in discrepancies within the literature, despite the use of similar animal models under similar experimental settings. Several semi-automatic approaches to analyze muscle histopathology currently exist [12–15]. However, their usage has not been widely adopted, likely due to the cost or the difficulty to implement them with some requiring basic programming skills.

Recently, a free, open-source software called CellProfiler has increasingly gained popularity and visibility in the field of automated image analysis, which provides a platform for the user to create customized pipelines for image analysis. CellProfiler is developed by the Carpenter Laboratory at the Broad Institute of Harvard and MIT that allows investigators with little prior bioinformatics knowledge to automate image analysis and collect large amounts of phenotypic data relatively easily [16, 17]. The goal of this work is to provide a free, easy-use, fast, and reliable pipeline for CellProfiler to analyze and quantify muscle histological properties using immunofluorescence images of muscle cross-sections.

## Implementation

### Mice

Mice (C57BL/10ScSn and C57BL/10ScSn-*Dmd*^*mdx*^/J) were maintained at The Ohio State University Laboratory Animal Resources in accordance with animal use guidelines. All animal studies were authorized by the Animal Care, Use, and Review Committee of the Ohio State University.

### Immunofluorescence staining of muscle cross-sections and imaging

Quadriceps muscles were collected from five male wild-type (WT) and five male *mdx* mice at the 8 weeks of age. Skeletal muscle tissues were mounted in Optimal Cutting Temperature (OCT) and frozen in liquid nitrogen cooled isopentane. Muscle cryosections were prepared using Leica CM3050S cryostat (Leica Biosystems, Buffalo Grove, IL, USA) at a thickness of 7 μm. The sections were fixed with 4% paraformaldehyde for 15 min at room temperature followed by two washes with PBS and 1 h incubation with blocking solution (5% bovine serum albumin) prior to overnight incubation at 4 °C with primary antibody against laminin α2 (ALX-804-190, 1:100, Alexis). The slides were then extensively washed with PBS and incubated with secondary antibodies (Alexa Fluor 488 goat anti-rat IgG, 1:500, Invitrogen) for 1 h at room temperature. Finally, the slides were mounted using VECTASHIELD® Mounting Medium with DAPI (Vector Laboratories, Inc.) and imaged with a × 20 lens in an inverted Nikon microscope (Nikon). A total of 70 non-overlapping images (approximately 250 muscle fibers per image for WT and 200 for *mdx*) were captured from 5 WT and 5 *mdx* mice (7 images per mouse) and saved in the ND2 file format with green channel for laminin and blue channel for DAPI. These images were also exported into the TIFF file format. Both ND2 and TIFF formats can be used as input images for CellProfiler.

### CellProfiler-facilitated automation of image processing

The stable version (2.2.0) of CellProfiler downloaded from the CellProfiler website (www.cellprofiler.org) and installed on a PC (Intel Xeon CPU E5–1620 v2 @3.70 GHz, 32.0 GB RAM, and 64-bit Windows 7 operating system) was used for the data processing in this manuscript. The current stable version is 3.0.0 at the time of this manuscript submission. CellProfiler is available for Windows, Mac and Linux. Java installation is required prior to installing CellProfiler. Users are encouraged to read the CellProfiler manuals (http://cellprofiler.org/manuals/) before testing the pipeline. The MuscleAnalyzer pipeline (ND2) for CellProfiler version 2.2.0 (Additional file 1) and 3.0.0 (Additional file 2), as well as the MuscleAnalyzer pipeline (TIFF) for CellProfiler version 3.0.0 (Additional file 3) are available online.

### Manual muscle fiber counting and CNF determination

To further validate the data generated by CellProfiler, muscle fiber counting, CNF percentage, CSA, and MFD were determined manually using Nikon NIS Elements software (version 4.3, Nikon). For CNF counting, two different classes were assigned for CNF and total fibers under count and taxonomy from manual measurement control window. To determine CSA and MFD, each individual muscle fibers were detected as object manually. The results were exported to Excel files. Total of seven non-overlapping images per section of each mouse were captured, and the percentage of CNF was determined.

## Results

### Muscle fiber and nuclei identification

**Fig. 1:**
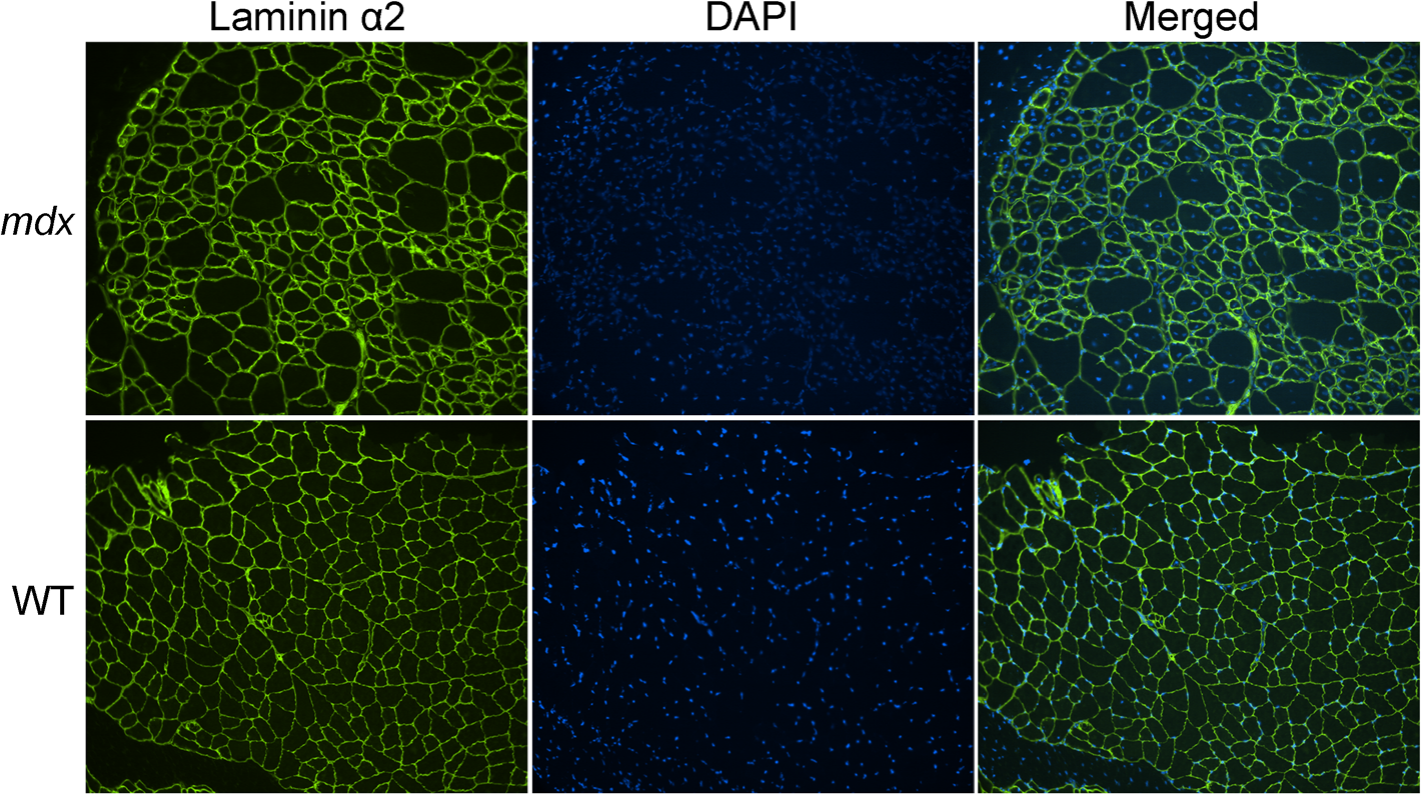
Representative immunofluorescence images of WT and *mdx* skeletal muscle sections stained with laminin α2 (green) and DAPI (blue)

**Fig. 2:**
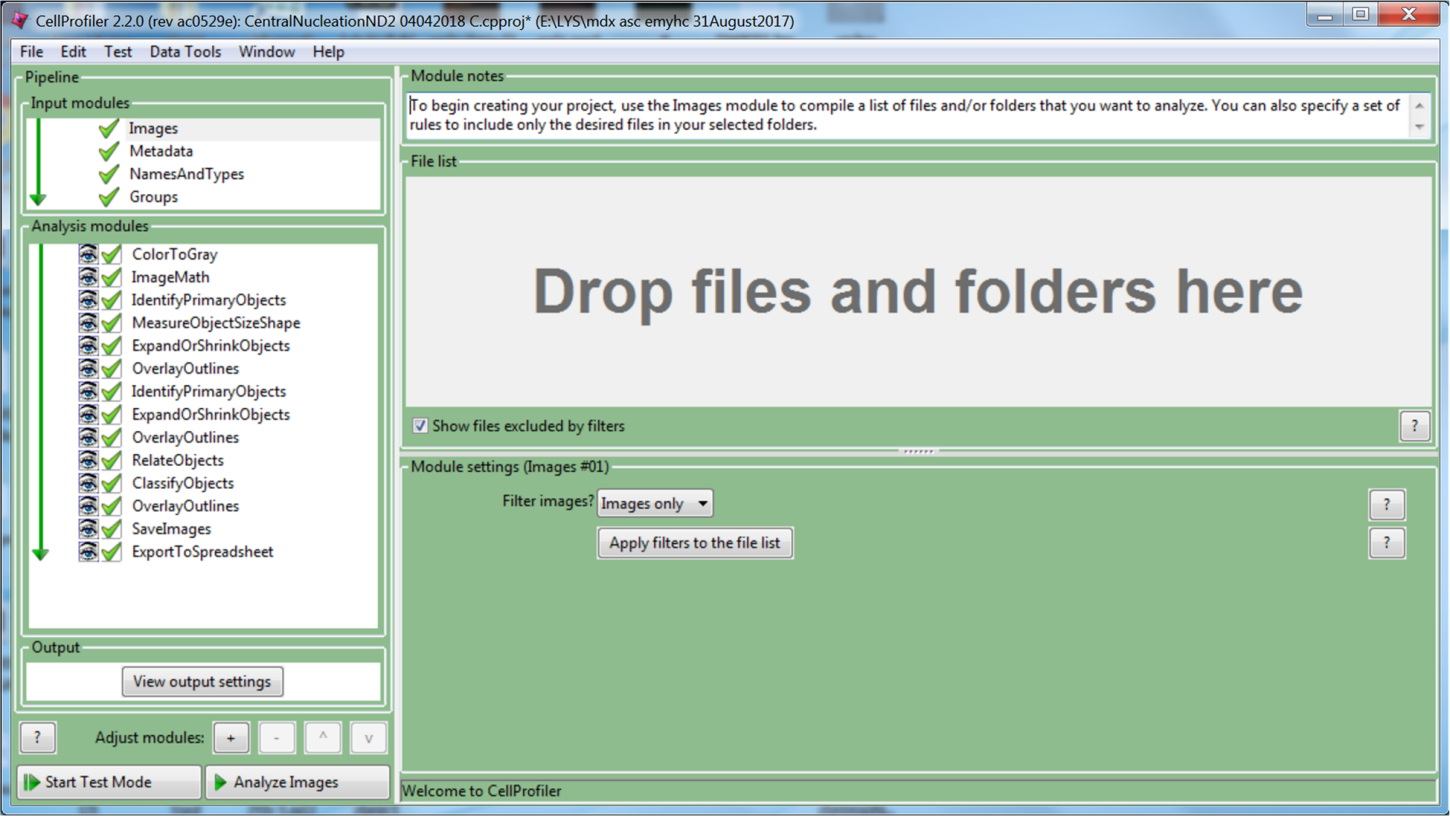
CellProfiler (version 2.2.0) interface. **a** The pipeline panel consists of input modules for data entry (image file or folder name, image type, and grouping of images). The analysis modules is the platform to build up the analysis pipeline from the module ‘ColorToGray’ to ExportToSpreadsheet,’ which can be inserted by clicking the ‘+’ sign below the pipeline panel

The laminin α2 (green) and DAPI (blue) co-stained muscle sections of WT and *mdx* mice were imaged and saved to ND2 files using the NIS-Elements Advanced Research software provided by Nikon (Fig. 1). CellProfiler supports a wide variety of image formats, including most of those used in imaging, by using a library called Bio-Formats (http://docs.openmicroscopy.org/bio-formats/5.7.0/supported-formats.html). In our initial test, we found that CellProfiler can directly analyze both ND2 files and TIFF files, and thus for all our following studies, we used ND2 files without prior conversion into TIFF files. Upon startup, the user is provided with an empty pipeline, which consists of Input modules, analysis modules, and output settings (Fig. 2). A typical CellProfiler workflow is summarized in the flowchart (Additional file 4). Basically, the images are loaded and then processed (e.g., cropping, illumination correction, object identification, object classification, measurements, and data output). A step-by-step video tutorial is provided to guide the implementation of MuscleAnalyzer pipeline (Additional file 5).

To demonstrate the process of automated imaging analysis using CellProfiler, we first loaded an *mdx* image in ND2 file format by dragging the file into the “File list” of the “Images modules” under “Input modules” (Fig. 2). The metadata of the image can be extracted using the “Metadata module.” You may also need to set up the correct image type using the “NamesAndTypes module” and assign a name to the image. Under the “Analysis modules,” individual analysis modules can be added.

**Fig. 3:**
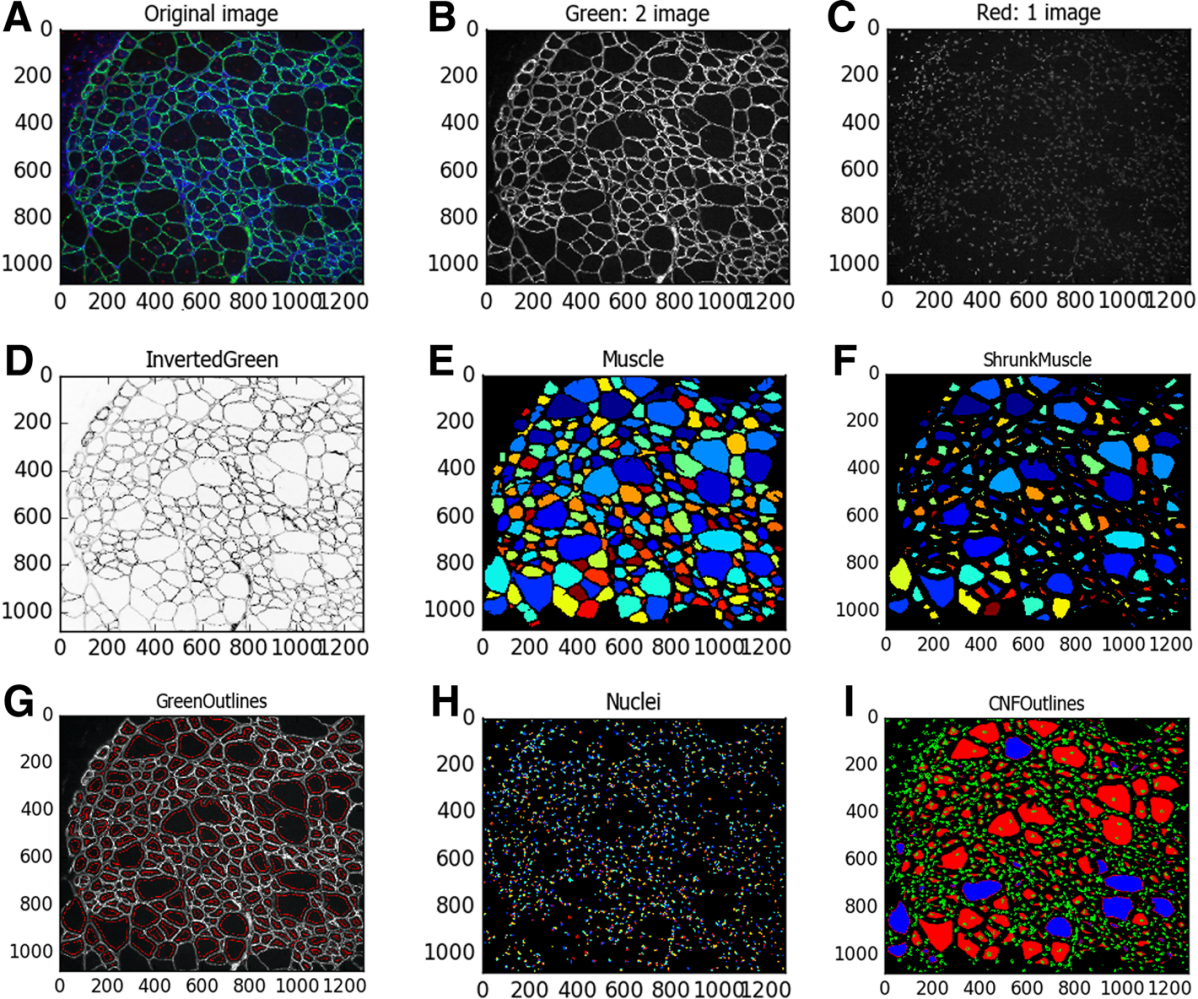
Sample image processing by CellProfiler. **a** Original RBG image of *mdx* muscle section stained with laminin α2 (green) and DAPI (blue). **b**, **c** Converted grayscale images of each channel after running the “ColorToGray” module. **d** Inverted green channel image. **e** Pseudo-colored image to show individual muscle fibers identified by using ‘IdentifyPrimaryObjects’ module. **f** The identified muscle objects were shrunk by seven pixels in order to determine if they contain central nuclei. **g** Outlines of identified muscle fibers were overlaid with original green channel image to illustrate the accuracy of muscle fiber identification. **h** Pseudo-colored image to show individual nuclei identified by using ‘IdentifyPrimaryObjects’ module. **i** The outlines of identified muscle fibers and nuclei were overlaid with original green channel image to illustrate the accuracy of classification with either normal (blue) or central nucleated muscles (red)

For analyzing ND2 color images (Fig. 3a), the first step is to split the color images into grayscale images for each channel using the “ColorToGray” module (Fig. 1). The green channel of laminin staining (Fig. 3b) will be used to identify the muscle fiber and blue channel of DAPI staining (Fig. 3c) will be used to identify the nuclei. The grayscale image of the green channel was then inverted using the “ImageMath” module (Fig. 3d). Next, the “IdentifyPrimaryObjects” module was used on the inverted image of the green channel to identify the muscle fiber. By setting the minimal and maximal diameter of muscle fibers, we can filter out any objects outside the diameter range. We used the “RobustBackground” method to threshold the image, and the key parameters such as threshold correction factor and size of smoothing filter for declumping are important to determine the reliability of muscle fiber identification. By adjusting these parameters, we were able to achieve a satisfactory result in identifying muscle fibers on our sample image (Fig. 3e). By applying the “MeasureObjectSizeShape” module here, we can obtain total muscle fiber numbers and the muscle fiber area, which can be exported into an Excel file later using the “ExportToSpreadsheet” module. The muscle fiber objects were shrunk by several pixels (in our sample image, we set the number of pixels to be 7) using the “ExpandOrShrinkObjects” module (Fig. 3f) in order to classify them as CNF or normal later. Using shrunk muscle fibers would ensure that these associated nuclei are located on the muscle edge or in the center. An overlay of the identified muscle fibers (red) with the original green channel image (gray) in Fig. 3g showed that the majority of the muscle fibers were correctly identified.

To identify the nuclei on the DAPI-stained nuclei image in the blue channel, we applied the “IdentifyPrimaryObjects” module again by using the “Automatic” threshold strategy. The smoothing filter size and maxima suppression distance were again important for faithfully detecting the nuclei. As shown in Fig. 3h, we were able to detect the nuclei on the sample image. For central nuclei classification, we again used the “ExpandOrShrinkObjects” module to shrink the nuclei object to a point to minimize the chance whereas the nuclei objects touching the border of muscle objects were incorrectly classified as central nuclei.

### Count CNF and normal muscle fibers

After having successfully identified the muscle fibers and the nuclei, our next step is to relate these two objects so that we can count the muscle fibers with or without central nuclei. For this purpose, we used the “RelateObjects” module to relate these two objects. The shrunk nuclei were used as the “child objects” while the shrunk muscle fibers as the “parent objects.” This allows us to count the number of child objects (shrunk nuclei) associated with each parent object (shrunk muscle fibers). After this step, the “ClassifyObjects” module can be applied to classify the shrunk muscle fibers with 0 (normal) or at least 1 nuclei (CNF). As shown in Fig. 3i, the CNF (red) and normal muscle fibers (blue) were correctly differentiated. At the end of the pipeline, “SaveImages” and “ExportToSpreadsheet” modules can be used to save images and export measurements to Excel files, respectively.

### Comparison between manual and semi-automated approaches

**Fig. 4:**
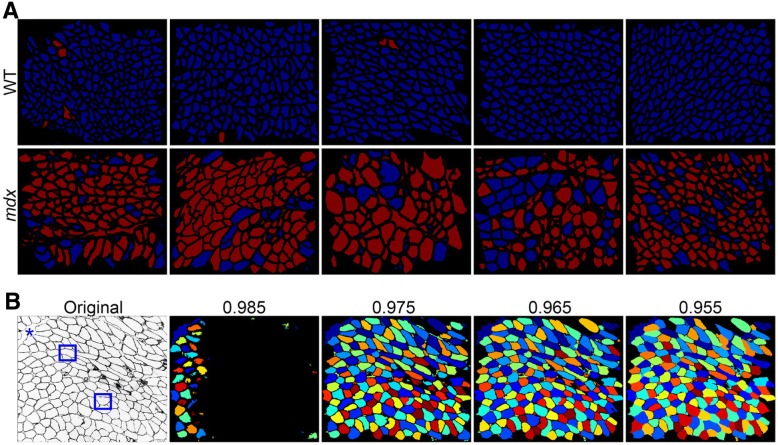
Examples of correctly and incorrectly processed muscle images. **a** Representative pseudo-colored images from all five WT and *mdx* mice after processed by CellProfiler. Red, centrally nucleated fibers; blue, normal fiber. **b** An incorrectly processed image with a large black area, which was due to a high threshold setting and can be corrected by lowering the threshold from 0.985 to 0.955. The blue stars showing the inter-fiber spaces that were mistakenly identified as muscle fibers; the blue boxes indicating individual muscle fibers that were mistakenly split into two

To compare the performance of the MuscleAnalyzer pipeline versus manual fiber counting, we captured 7 images of randomly chosen non-overlapping regions per muscle section from WT and *mdx* mice (5 each), resulting in a total of 70 images to be analyzed. A quick examination of these 70 images found that three images had large area of artifact staining in blue channel and were excluded from the analysis. We thus analyzed the 67 images (Additional file 6) by either CellProfiler or the traditional manual approach. It took about 11 min for CellProfiler on a PC (Intel Xeon CPU E5-1620 v2 @3.70 GHz, 32.0 GB RAM, and 64-bit Windows 7 operating system) to complete all these 67 images. One image from each of the 5 WT and 5 *mdx* muscles were shown in Fig. 4a. Clearly, the majority of the WT muscle fibers were identified as normal (blue) while the majority of the *mdx* muscle fibers were identified as CNF (red). Careful examination of the analyzed images found three were not correctly processed with a large black area (Fig. 4b). This was due to the “threshold correction factor” being set at too high. By lowering down this value from 0.985 to 0.975, we can recover the missing muscle fibers on this particular image (Fig. 4b). Careful comparison of the pseudo-colored muscle fiber image (the right image) with the original laminin-stained image shown on the left (Fig. 4b) showed that several mistakes were made by CellProfiler. Some inter-muscle fiber regions were identified as muscle fibers (Fig. 4b, blue star). In addition, some muscle fibers were split into two (Fig. 4b, blue boxes). However, most muscle fibers were correctly identified.

**Fig. 5:**
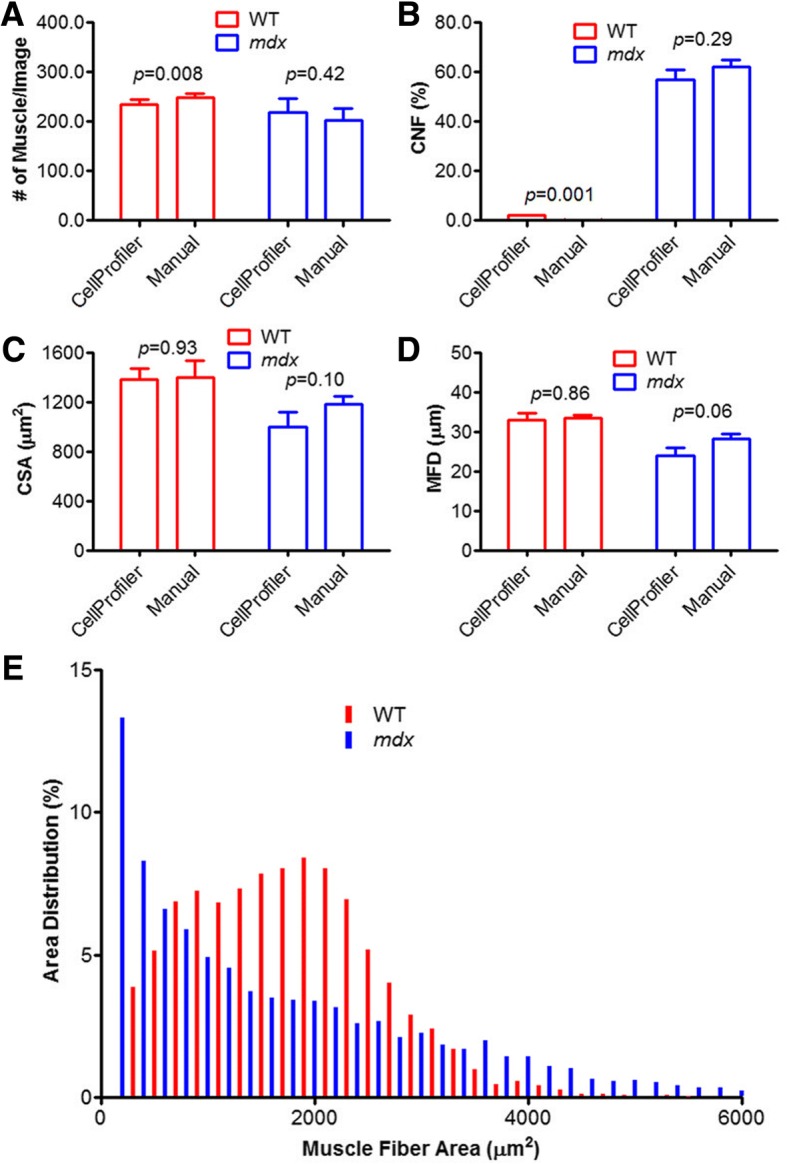
Quantitative measurements of the 67 images processed manually or by CellProfiler. **a** The average number of muscle fibers per image. **b** The percentage of CNF. **c** CSA measurements. **d** MFD measurements determined by CellProfiler or the manual approach. **e** Size distribution of WT and *mdx* muscle fibers generated from the CSA data produced by CellProfiler

We also analyzed these 67 images by the manual approach. It took an experienced investigator roughly 3 h to complete the task. The average number of muscle fibers identified per image with CellProfiler was similar to that counted by the manual approach in *mdx* samples; however, there was about 6% less of muscle fibers identified by CellProfiler than the manual approach for the WT samples (Fig. 5a). The calculated CNF percentage was again fairly comparable between the two approaches for *mdx* samples (56.7 by CellProfiler vs 61.8 manually, *p* = 0.29) (Fig. 5b), indicating that the CellProfiler can be used to automate the process for muscle immunofluorescence image analysis. It is of note that CellProfiler obtained significantly more CNFs than the manual approach did in WT samples (1.9 by CellProfiler vs 0.1 manually, *p* = 0.001) (Fig. 5b), however, these numbers are still within the normal range of healthy sample variations. Moreover, we also compared these two approaches for the measurement of CSA and MFD using three independent images per genotype. As shown in Fig. 5c, d, CellProfiler obtained very similar measurements as the manual approach; however, the time used by CellProfiler to complete the same task was only a small portion of that used by the latter. Finally, we used CellProfiler to automate the measurement of CSA for all 67 images to derive the fiber size distribution. Consistent with the muscular dystrophy phenotype, *mdx* muscle showed an increase in both very small (regenerative) and very large (hyper-contracted) muscle fibers, while WT muscles showed a more even distribution (Fig. 5e).

## Discussion

In this study, we compiled a pipeline termed MuscleAnalyzer for CellProfiler to automatically process immunofluorescence images of muscle cross-sections stained with laminin α2 and DAPI. Laminin α2 staining was used to facilitate the determination of individual muscle fibers while DAPI staining was used to label cell nuclei. The parallel comparison with MuscleAnalyzer and manual approach showed that the MuscleAnalyzer pipeline can provide relatively accurate measurements of muscle features such as CNF percentage, fiber diameters, and fiber size distribution with minimal efforts and time. This should aid the pathophysiological studies of muscle diseases and evaluation of therapeutic impact.

The most critical steps involve the identification of muscle fibers and nuclei, and then classification of muscle fibers into CNF or normal through correlation of nuclei with muscle fibers. CellProfiler uses the term “object” as a generic term to refer to an identified feature (for example, nuclei and muscle fibers) in an image. Nuclei are more easily identifiable due to their more uniform morphology, high contrast relative to the background with DAPI staining, and good separation between adjacent nuclei. However, muscle cells often have irregular morphology, varying sizes, uneven and more diffused staining patterns, making them much more challenging to identify than nuclei [12, 18]. Moreover, muscle cells often touch their neighbors making it harder to delineate the cell borders. To use the “IdentifyPrimaryObjects” module to identify either nuclei or muscle fibers, the ND2 images should be converted to grayscale images for the corresponding channels. The laminin-stained channel needs to be inverted using the “ImageMath” module. Object identification (segmentation) is performed through image thresholding, recognition and division of clumped objects, and removal or merging of objects on the basis of size or shape [16]. Therefore, it is important to test the thresholding parameters for correct segmentation. The “Test Mode” provided by CellProfiler makes it convenient to test individual modules of the pipeline before final batch analysis of a large set of images.

For laminin-stained muscle sections, we found that the “Global” strategy of thresholding, which calculates a single threshold value based on the unmasked pixels of the input image and use that value to classify pixels above the threshold as foreground and below as background, and the “RobustBackground” method for finding thresholds automatically, provided the best results in muscle fiber identification. The “RobustBackground” method assumes that the background distribution approximates a Gaussian by trimming the brightest and dimmest 5% of pixel intensities, and then calculates the mean and standard deviation of the remaining pixels, and the threshold as the mean + 2 times the standard deviation [16, 19]. The threshold can be further adjusted either upwards or downwards through multiplying it by the “threshold correction factor.” The strategy that we used to classify the CNF is to relate the nuclei and muscle fibers using the “RelateObjects” module after shrinking muscle fibers by several pixels and nuclei to a point. This strategy appears to be robust; however, the number of pixels to be shrunk for muscle fibers need to be empirically determined.

Three common errors associated with identification of muscle fibers include (1) some inter-muscle fiber spaces and blood vessels were mistakenly counted as muscle fibers due to the fact that the laminin staining on the surrounding muscle fibers formed closed compartments; (2) some muscle fibers were merged due to the difficulty in correctly finding the borders of the touching muscle fibers; and (3) some muscle fibers were split into smaller ones due to high background intracellular staining. Carefully adjusting the parameters for thresholding can greatly minimize the rate of these errors but does not seem to completely get rid of them. It is worthy to test if adding a third staining of muscle fibers such as collagen (to label inter-muscle fiber regions) and muscle-specific cytoplasmic proteins (i.e., desmin and α-actinin, to label muscle fibers) could help to further reduce the errors in the future.

Several other semi-automatic analysis tools have been reported [12–15]. We have attempted to test and compare these tools with CellProfiler and found CellProfiler is relatively easy to implement. We did not test all these tools because they are either not available on internet or purchase is required. From the original reference, it appears that MuscleQNT can quantify only CSA and fiber distribution, while the MuscleAnalyzer pipeline can obtain CNF, MFD, CSA, and potentially other more parameters with some modifications. We were able to download, install, and test the standalone SMASH program, unfortunately we were unable to export the data to Excel files at the end. Moreover, SMASH can only analyze the images one-by-one, while CellProfiler can analyze the data in a batch format, enabling full automation. Finally, CellProfiler provides a free and flexible platform to a wide range of users in performing image analysis, which has been cited more than 6000 times. The established pipelines are easy to share among the research community allowing fast improvement and increased scientific reproducibility.

There are several limitations for the current version of our MuscleAnalyzer pipeline. First, it does not incorporate the function to manually correct the wrongly identified muscle fibers. However, our initial study with the 67 images showed that the error for muscle fiber identification was less than 10%. In a fully automation setting to analyze a large number of images, such an error rate does not appear to affect the conclusion of CNF, CSA, and MFD, particularly in diseased muscles. Second, we have not incorporated the function to analyze other useful parameters for muscle biology, such as satellite cells, muscle fiber types, and muscle fibrosis. Future improvements can be made to incorporate these functions. Last but not least, it is important to acquire high quality images in order for the MuscleAnalyzer pipeline to accurately identify muscle fibers and nuclei. Tissue tearing/folding and freezing artifacts during tissue section preparation should be minimized.

## Conclusions

Taken together, the MuscleAnalyzer pipeline for CellProfiler allows rapid and accurate batch analysis of skeletal muscle cross-sectional immunofluorescence images. Although we only test it for CNF, CSA, and MFD, one can envision it would allow for quantification of other traits of skeletal muscle such as characterization of muscle satellite cell, muscle fiber type, necrosis and fibrosis with minor modifications of the pipeline. This should aid the unbiased pathophysiological studies of muscle diseases and evaluation of therapeutic impact.

## Availability and requirements

Project name: MuscleAnalyzer pipeline for CellProfiler version 2.2.0 and 3.0.0.

Project homepage: N/A.

Operating system: Platform Independent.

Programming language: Python.

Other requirements: MuscleAnalyzer pipeline requires CellProfiler which is freely available from CellProfiler (http://cellprofiler.org/) developed by the Carpenter Lab at the Broad Institute of Harvard and MIT. Java installation is required prior to installing CellProfiler.

License: CC-BY.

Any restrictions to use by non-academics: None.

## Additional files

Additional file 1: MuscleAnalyzer pipeline (ND2) for CellProfiler version 2.2.0. (CPPIPE 19 kb)

Additional file 2: MuscleAnalyzer pipeline (ND2) for CellProfiler version 3.0.0. (CPPIPE 17 kb)

Additional file 3: MuscleAnalyzer pipeline (TIFF) for CellProfiler version 3.0.0. (CPPIPE 17 kb)

Additional file 4: Flow chart illustrating the sequence of image processing with CellProfiler. (PPTX 41 kb)

Additional file 5: A step-by-step video tutorial for image analysis using MuscleAnalyzer pipeline in CellProfiler 3.0.0. (MOV 120693 kb)

Additional file 6: Sample image dataset. (RAR 358466 kb)
