# Supplementary material for: Automated muscle histopathology analysis using CellProfiler
Source: Skelet Muscle. 2018 Oct 18;8:32. doi: 10.1186/s13395-018-0178-6 (PMC6193305; doi:10.1186/s13395-018-0178-6)
Supplement: Supplementary file 4 — Flow chart illustrating the sequence of image processing with CellProfiler. (PPTX 41 kb) [file 13395_2018_178_MOESM4_ESM.pptx]

## Slide 1
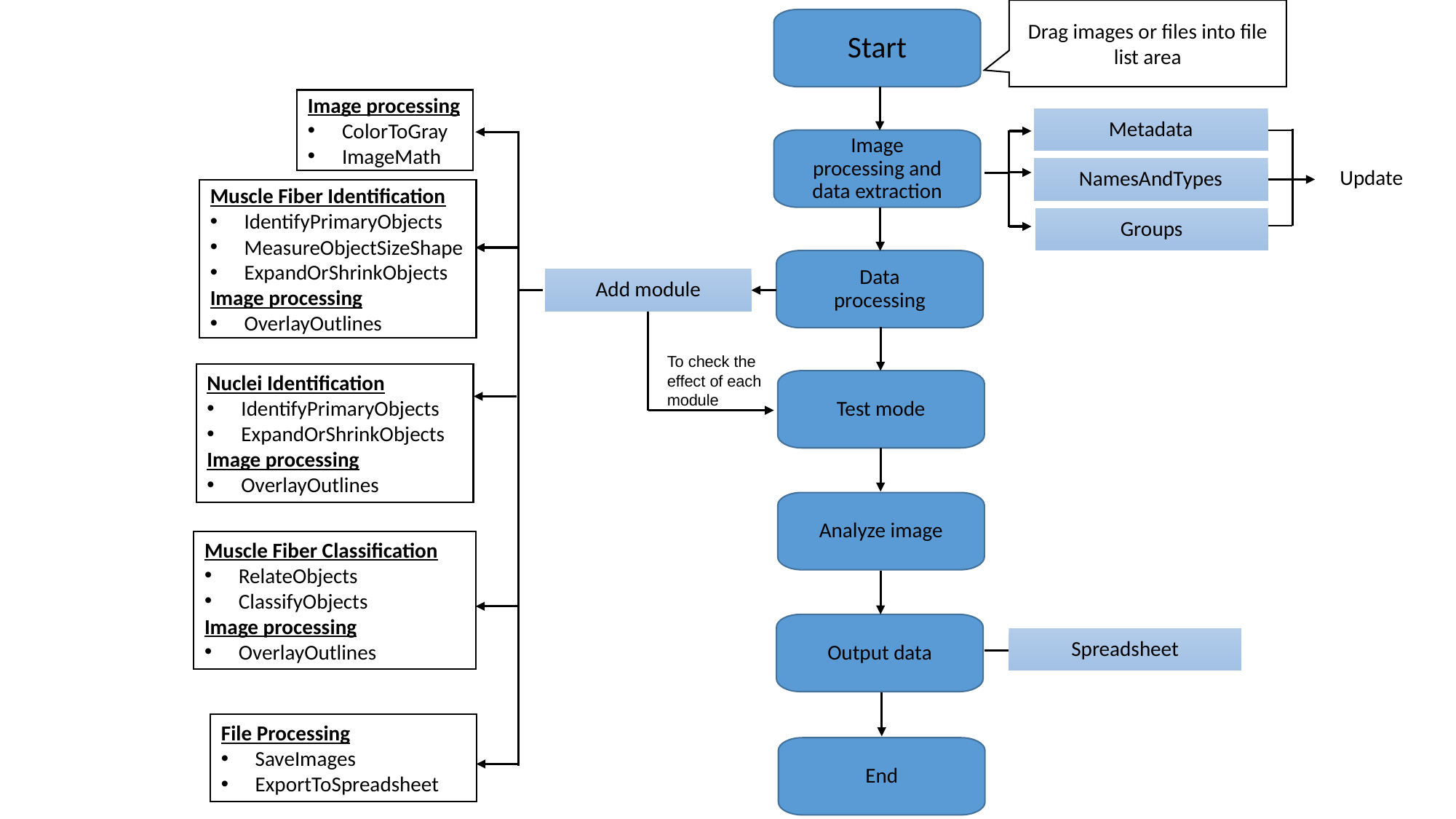

Drag images or files into file list area
Start
Image processing
ColorToGray
ImageMath
Metadata
Image processing and data extraction
Update
NamesAndTypes
Muscle Fiber Identification
IdentifyPrimaryObjects
MeasureObjectSizeShape
ExpandOrShrinkObjects
Image processing
OverlayOutlines
Groups
Data processing
Add module
To check the effect of each module
Nuclei Identification
IdentifyPrimaryObjects
ExpandOrShrinkObjects
Image processing
OverlayOutlines
Test mode
Analyze image
Muscle Fiber Classification
RelateObjects
ClassifyObjects
Image processing
OverlayOutlines
Output data
File Processing
SaveImages
ExportToSpreadsheet
End
Spreadsheet
